# Two dimensional diffusion-controlled triplet–triplet annihilation kinetics†
†Electronic supplementary information (ESI) available. See DOI: 10.1039/c9sc00957d


**DOI:** 10.1039/c9sc00957d

**Published:** 2019-07-04

**Authors:** Grégoire C. Gschwend, Morgan Kazmierczak, Astrid J. Olaya, Pierre-François Brevet, Hubert H. Girault

**Affiliations:** a Laboratoire d'Électrochimie Physique et Analytique , École Polytechnique Fédérale de Lausanne , Rue de l'Industrie 17 , CH-1951 Sion , Switzerland . Email: hubert.girault@epfl.ch; b École Normale Supérieure , Département de Chimie , PSL Research University , 75005 , Paris , France; c Institut Lumière Matière , UMR CNRS 5306 , Université Claude Bernard Lyon 1 , Campus LyonTech La Doua , 10 Rue Ada Byron , 69622 Villeurbanne Cedex , France

## Abstract

Diffusion controlled chemical reactions are usually observed in three dimensional media. In contrast, planar bimolecular reactions taking place between reagents adsorbed at a soft interface are two-dimensional and therefore cannot be studied within the same formalism. Indeed, soft interfaces allow the adsorbed species to freely diffuse in a liquid-like manner. Here, we present the first experimental observation of a diffusion-controlled reaction in an environment that is planar at the ångström scale. By means of time-resolved surface second harmonic generation, an inherently surface sensitive technique, we observed that the kinetics of the diffusion of the reagents in the plane decreases as the surface concentration of adsorbed species increases. This is of course not the case for bulk reactions where the rates always increase with the reactant concentration. Such changes in the kinetics regime were rationalised as the evolution from a regular 2D free diffusion regime to a geometry-controlled scheme.

## Abstract 2

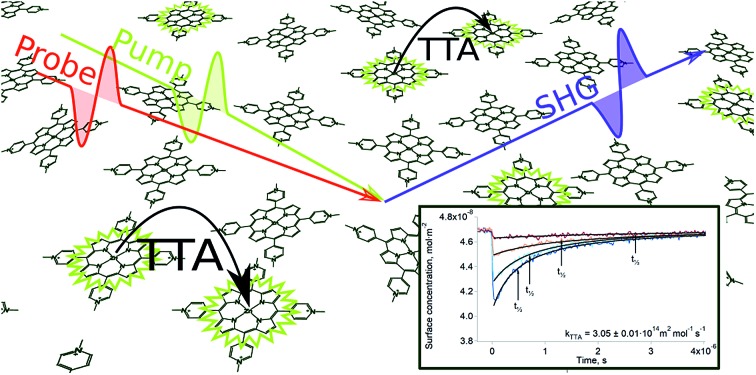
We show with time-resolved second harmonic generation and molecular mechanics simulations that the kinetics of a two-dimensional triplet–triplet annihilation reaction at the liquid–liquid interface is affected by molecular crowding.

## Introduction

Following the very early work of Benjamin Franklin, studying the spreading of a spoon of oil on water, Lord Rayleigh was able to show that this spreading forms a molecular film and from simple geometric considerations was able to measure the thickness of a molecule. Pockels^1^ and later Langmuir^2^ improved the methodology to study molecules on a planar soft interface. So, if purely two dimensional monolayer molecular films have been observed for more than a century, very few studies have been concerned with reaction kinetics within these monolayers, and were often limited to unimolecular reactions.^3^,^4^

The interface between two immiscible liquids is a medium with physical properties that significantly differ from that of the two bulk phases. It has been shown, for instance, that in such systems the polarity of the interface is the average of that of the two liquids^5^–^7^ and that it changes on a subnanometer length scale across the interface.^8^ Likewise, the viscosity of the interface is different from that of the contacting solvents, which leads, for example, to peculiar non-radiative de-excitation pathways of adsorbed malachite green molecules.^9^ The reduced dimensionality of the interface is another difference when compared to the bulk solutions. The consequences of the dimensionality on the way a particle trapped at an interface can explore its surrounding space are commonly treated using random walks. One of the well-known results of this approach is that two-dimensional walks are recurrent while higher dimensional walks are transient. Such models, applied to simulated chemical reactions, have shown surprising dimension-dependent kinetics, with consequences on the survival probability over time of the reacting particles,^10^,^11^ as well as segregation effects of the reagents.^12^–^14^ Consequently, the experimental study of diffusion-influenced chemical reactions occuring within the plane of soft surfaces requires a dedicated approach. Specific models designed to treat such systems showed that compared to three-dimensional cases, two-dimensional rate constants – or coefficient, as they are no longer constant^15^ – never reach an asymptotic value, implying that the reactions do not formally attain a steady state.^15^–^18^ As demonstrated by Bénichou *et al.*, many diffusion schemes actually belong to a same universality class,^19^ and an important parameter to classify the diffusion processes is the compact *versus* non-compact exploration schemes. These schemes depend on the dimensions of the walk and of the surrounding medium. Such peculiar reactions are not only interesting at the fundamental level but have also practical applications for chemical reactions taking place in biological membranes.^20^–^22^ However, there are only a few examples where this formalism has been applied to two-dimensional media,^23^–^25^ and these examples measured reaction kinetics in modified lipid films which, although more similar to biological systems, are not truly two-dimensional as the motion of the probes are, to some extent, independent of the lipid motion.

The present work reports the observation of triplet–triplet annihilation (TTA) of the first excited triplet state of a zinc porphyrin (tetrakis(1-methyl-4-pyridinio)porphyrin chloride, ZnTMPyP) at the interface between two immiscible liquids (α,α,α-trifluorotoluene and water), which can be considered as a truly two-dimensional bimolecular reaction. Triplet–triplet annihilation is a photochemical reaction in which two excited triplets react, promoting one of the triplets to a higher singlet state and de-exciting the other one, the overall equation being:

1 T_1_ + T_1_ → S_1_ + S_0_

The decays measured by time-resolved surface second harmonic generation (TR-SHG) show that the excited triplet population half-life at the interface is four orders of magnitude shorter than in the bulk at similar concentrations. This behaviour is due to the adsorption of the molecules at the interface that locally increases their density, reaching values significantly higher than in the bulk. Thus, at the interface, the average distance between two porphyrins is much shorter than in solution. The close packing of the molecules has two opposing effects: it favours the TTA pathway between neighbour molecules, but at the same time, hinders the diffusion between diffusing molecules and consequently lowers the reaction kinetics. To the best of our knowledge, this is the first example of a diffusion-controlled reaction taking place in a medium that is truly two-dimensional at the ångström scale, indeed, the *z*-coordinate of the adsorbed molecules does not vary more than 0.8 nm (see S2†). Furthermore, we show that a chemical reaction in a plane can progressively evolve with the surface concentration from a normal diffusion regime to a slower transport regime impacting its kinetics. Thus, these results present an experimental confirmation that compactness is a key parameter to characterise reaction kinetics.

## Results and discussion

### Time-resolved surface second harmonic generation

**Fig. 1:**
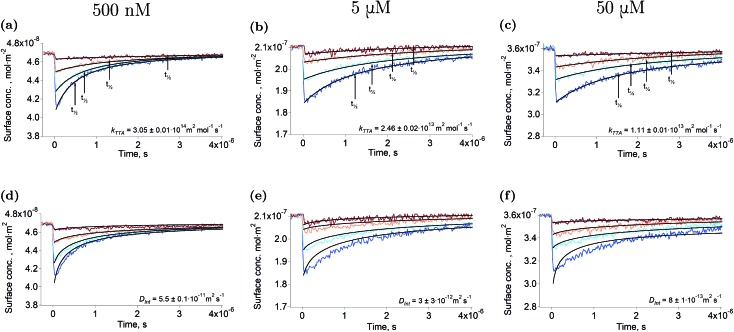
Triplet state decay and kinetic fitting. Ground state recovery of ZnTMPyP after excitation at 565 nm at various intensities (from brown to blue: ∼125 nJ, ∼250 nJ, ∼500 nJ, ∼1 μJ) under N_2_ atmosphere. The bulk concentrations are: 500 nM (12% coverage): (a, d), 5 μM (58% coverage): (b, e) and 50 μM (93% coverage): (c, f). The data were fitted using eqn (2) in order to obtain the rate constant (a–c), and using the model of Razi-Naqvi^16^ and Owen^26^ in order to obtain the diffusion coefficients (d–f). The estimation of the surface concentration is explained in the ESI.†

Fig. 1a–c show the second harmonic intensity of a 880 nm pulse – resonant with the Soret band of ZnTMPyP – as a function of the time delay with the excitation pulse at 565 nm, at various surface concentrations and excitation intensities (the surface concentration determination is explained in “Experimental part” and a complete description of the setup is avaible in the ESI†). No second harmonic signal specific to the triplet state of ZnTMPyP was observed in the experimental conditions by varying the probe wavelength from 400 nm to 500 nm. Thus, the decays presented in Fig. 1 correspond to the repopulation of the ground state and therefore, the depletion is directly proportional to the concentration of the triplet excited state. The triplet concentration was controlled by varying the excitation intensity. It has to be noted that the excitation pulse actually populates the first excited singlet, however, due to the high intersystem crossing quantum yield of this porphyrin (90%),^27^ and because this phenomenon is fast, only the lowest triplet can be detected in the time scale of this experiment. The traces presented in Fig. 1 show that the half-life of ZnTMPyP* (triplet state of ZnTMPyP) at the liquid–liquid interface is of the order of hundreds of nanoseconds to few microseconds. These half-lives are short compared to values in the bulk at similar concentrations (∼3.8 ms), indicating that the interface is remarkably different. Experiments conducted at the interface between water and 1,2-dichlorethane (DCE) or dichloromethane (DCM) produced identical results, meaning that the solvent is not responsible for the fast decay. Another explanation of a shorter half-life could be aggregation of the porphyrin. Indeed, if dimerisation of ZnTMPyP occured at the interface, one could observe the decay of a supramolecular assembly instead of that of ZnTMPyP*. For instance, Nagatani *et al.* observed a red shift of the second harmonic spectrum of ZnTMPyP, which was tentatively explained as J-aggregation.^28^ However, the spectra that we measured could be fitted with only one Gaussian peak, suggesting that there is only one species at the interface (see Fig. S3†). Furthermore, when aggregation was forced in solution by addition of an hydrophobic counter-ion, no excited state could be detected in the tens of nanosecond time scale. This observation further supports that only monomers are present at the interface.

**Fig. 2:**
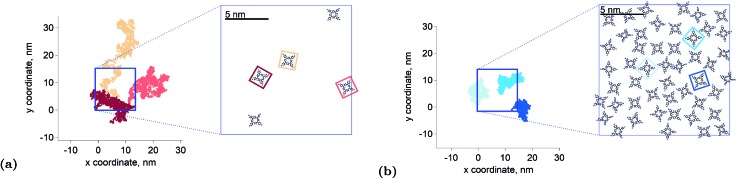
Illustration of the interface population and dynamics. Examples of trajectories and corresponding interfacial population from the molecular mechanics simulations at 10 percent (a) and 100 percent (b) surface coverage. At high concentration the molecules explore a smaller part of the interface. The blue squares show the limits of the simulation box, solvent molecules and counter ions have been removed for clarity. The trajectories are plotted without periodic boundary conditions.

The arrows on the traces presented in Fig. 1 show the actual half-lives of the excited porphyrin populations. It can be seen that these times are inversely proportional to the initial triplet concentrations, which were controlled by varying the excitation intensities. Such a behaviour is typical of a second order reaction assuming only a simple kinetic control. Indeed, the kinetics of the TTA can be described by the following equation:2
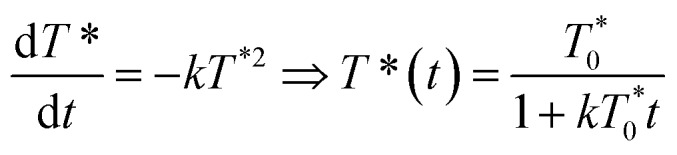
where T^*^(*t*) is the concentration of porphyrins in the triplet state at time *t*, 
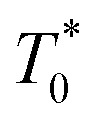
 the triplet concentration at *t* = 0 and *k* is the TTA rate constant. Thus, the half-life is: 
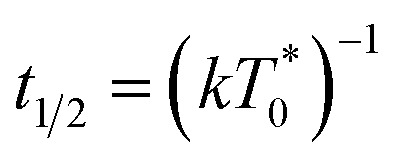
. The fact that the kinetics traces presented in Fig. 1 follow this trend further support the conclusion that the phenomenon responsible for the shorter lifetime at the interface is indeed TTA. Because of the time resolution of our setup (∼20 ns), any events taking place on and below this time scale cannot be probed. However, it is consistent with the TTA mechanism to suppose that all the excited porphyrins in close contact annihilate in the first tens of nanoseconds, leaving only a population of triplets too separated to react and whose reactivity is therefore limited by mass transport. Fig. 2 presents pictures of the simulated systems at 10 percent (Fig. 2a) and 100 percent (Fig. 2b) surface coverages, as well as typical trajectories of the adsorbed molecules. These graphs illustrate the change of surface compactness with the bulk concentration and its influence on the diffusion. Indeed, at full coverage the trajectories are more hindered and compact, implying a less efficient exploration of the interface. By contrast, at low surface coverage the molecules explore a larger portion of the interface.

### Fitting of the spectroscopic results

In a first simple kinetic approach, the rate constant of the TTA at the liquid–liquid interface can be obtained by fitting the results presented in Fig. 1 with eqn (2). The first-order decay can be neglected as its time scale is four orders of magnitude longer than that of the experiment. The rate constants thus obtained are presented in Table 1. It appears that the TTA rate constant is smaller at higher porphyrin concentrations, when the interface coverage increases. This observation suggests that the surface compactness changes the kinetics of the reaction. Such an effect of the compactness of the reaction medium on the kinetics has already been observed in the bulk, both experimentally and theoretically for biologically relevant systems,^29^,^30^ introducing the concept of geometry-controlled kinetics.^19^ In such systems, the large concentration of macromolecules or the presence of obstacles are thought to impede the diffusion of the reacting species, leading to anomalous diffusion and impacting therefore on the kinetics of the reactions. The hypothesis made in the present report is that at high surface coverages the adsorbed molecules in their ground state act as obstacles to the diffusion of the excited triplets, limiting their diffusion and therefore the triplet–triplet annihilation. We propose to rationalise the change of kinetics with the crowding of the interface by extracting the lateral diffusion coefficients of ZnTMPyP from the kinetic traces and by simulating the diffusion of the porphyrins at the liquid–liquid interface by molecular dynamics.

**Table 1:** Triplet–triplet annihilation rate constants and diffusion coefficients. The TTA rate constants have been obtained by fitting of the data with the eqn (2), while for the diffusion coefficients the eqn (2) and (4) were used. The relative surface coverages are given for each bulk concentrations (maximum surface concentration: 3.6 × 10^–7^ mol m^–2^). At high surface coverage the experimental diffusion coefficients are no longer in agreement with the simulated values. *k*_TTA_ (10^13^ m^2^ mol^–1^ s^–1^), *D* (10^–11^ m^2^ s^–1^)

*C*_Int_	500 nM (12%)	5 μM (58%)	50 μM (93%)
*k*_TTA_	30.5 ± 0.1	2.46 ± 0.02	1.11 ± 0.01
*D*_Int_	110 ± 10	0.3 ± 0.3	0.08 ± 0.01
*D*_Sim_	240 ± 60	230 ± 20	180 ± 20

As independently demonstrated by Razi-Naqvi^16^ and Owen,^26^ due to the two-dimensional nature of the interface between the organic and aqueous phases, conventional diffusion-influenced quenching equations like that of Smoluchowski cannot be directly applied to the present case, as they are derived in spherical coordinates, that is, for three-dimensional systems. The above-mentioned authors have thus developed a model based on the Smoluchowski approach, but derived in cylindrical coordinates, that yields an expression for the apparent time-dependent rate constant of diffusion-controlled reactions. In this model, the time-dependent diffusion rate parameter is given by:3
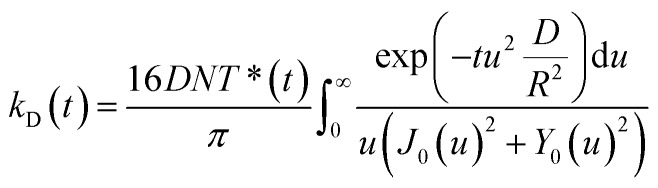
where *D* is the diffusion coefficient, *R* the reaction radius, T*(*t*) the time-dependent triplet concentration, *J*_0_(*u*) and *Y*_0_(*u*) the zero order Bessel functions of the first and second kind and *u* a dummy integration variable. In the present article, we use the same expression for the rate parameter but we insert it in the differential eqn (2), which yields:4
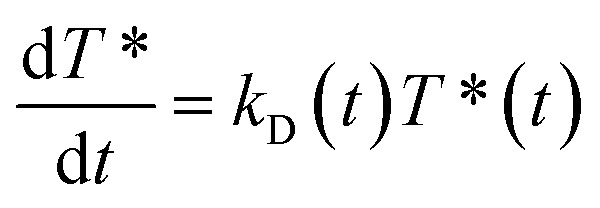



The resulting equation no longer has an analytical solution and therefore, the differential equation and the integral in eqn (4) were solved numerically in order to make the fitting. We used value of 1.5 nm for the reaction radius, which is the approximate size of the core of ZnTMPyP and is consistent with the mechanism of the TTA that involves the transfer of electrons and therefore orbital overlap of the reacting species. A detailed description of the model used and of the fitting procedure are presented in the ESI.† The obtained diffusion coefficients are presented in Table 1. At low surface concentrations, these values have orders of magnitude that could be expected for such systems and in concordance with the simulated diffusion coefficients. Nevertheless, the two-dimensional model does not appropriately fit the data at large surface coverage, and the resulting diffusion coefficients are far from the simulated values. As this model essentially relies on the resolution of the Fick diffusion equation in two-dimensions, we conclude that the observed discrepancies between the data and the model imply that in such conditions the diffusion of the species is no longer Fickian, which support the hypothesis of anomalous diffusion.

### Molecular mechanics simulations

Anomalous diffusion is characterised by a non-unity power law scaling of the mean squared displacement of a particle with time:^32^,^33^

5 〈((*x*_*i*_(*t*) – *x*_*i*_(*t*_0_))^2^〉 = = *D*_*i*_*t*^*d*^

**Fig. 3:**
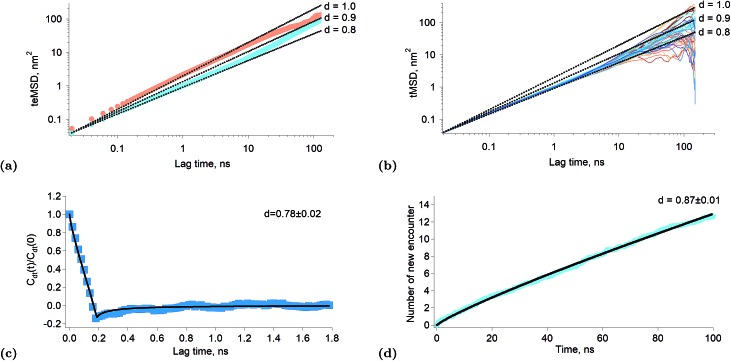
Results of simulated interfacial diffusion. (a) Time-averaged ensemble-average mean square displacement of the porphyrin simulated over 150 ns at 10 percent coverage (brown) and full coverage (blue). The black lines indicate scaling proportional to *t*^0.8^, *t*^0.9^ and *t*. (b) Individual time-averaged mean squared displacement of the porphyrin at full surface coverage. The black lines indicate scaling proportional to *t*^0.8^, *t*^0.9^ and *t*. (c) Displacement autocorrelation function calculated according to Jeon *et al.*^31^ The result is consistent with a fractional Langevin equation dynamics as shown by the fitting. The time interval was set to 200 ps. (d) Simulated average number of new encounter for a poprhyrin at full surface coverage as a function of time. The black line is a fitting with a power law.

When the exponent of the time variable is less than one, the diffusion regime is said to be sub-diffusive. Fig. 3a shows the time-averaged ensemble-averaged mean squared displacements (teMSD) of the porphyrin simulated by molecular dynamics over 150 ns at 10 percent and 100 percent surface coverages. These results show that the diffusion of the porphyrins at full surface coverage scales like *t*^0.8^ in the first tens of nanoseconds, while at longer times the system recovers a linear scaling. In contrast, the dependence is always linear at low surface coverage. Thus, the transport regime becomes sub-diffusive as the surface concentration of adsorbed porphyrins increases. Although anomalous diffusion is observed only in the time scale of few tens of nanoseconds, this still has consequences at every moments of the reaction because the transient regime is independent of the starting time of the experiment. Indeed, the first tens of nanoseconds of transport are abnormal independently of the absolute starting time of the observation. Thus, the overall reaction and equilibrium properties of a system can be affected by a transient anomalous regime.^34^ Furthermore, it can be seen in Fig. 3a that the mean squared displacement of a porphyrin after ∼30 ns is ∼20 nm^2^, which means a displacement of ∼4.5 nm. Such diffusion distances still allow triplets to react even at rather low surface concentrations, implying that indeed the sub-diffusion regime impacts on the kinetics of the tails of the traces presented in Fig. 1f.

There are different mechanisms underlying a sub-diffusive regime, like diffusion in fractal environments^15^ or in crowded or obstructed media.^12^ The latter mechanisms are usually treated using the continuous time random walk (CTRW) or the fractional Brownian motion (FBM) formalisms.^14^ One of the main consequences of the CTRW model is the weak ergodicity breaking, that is that the time and ensemble averages of thermodynamic quantities are no longer identical.^35^ Simulations of lipid bilayers have shown however that such systems were better described by fractional Langevin equation dynamics (FLE) because these systems were found to be ergodic.^31^Fig. 3b shows the time-averaged mean squared displacement (tMSD) of the individual porphyrins adsorbed at the liquid–liquid interface at full surface coverage. It appears that the sublinear scaling of the teMSD is also observed with the tMSD. This proves that there is no ergodicity breaking and that alike lipid bilayers the system is governed by FLE dynamics. This conclusion is also supported by the calculation of the displacement autocorrelation function (Fig. 3c) which again corresponds to what can be expected from FLE dynamics.^31^ A subdiffusive motion described by a FLE suggests the presence of a memory effect in the motion of the particles. Thus, it appears that as the interfacial concentration increases, the displacements of the porphyrins become correlated in time, impacting therefore on their reactivity.

## Discussion

Different models have been derived to analyse the diffusion-controlled reactions using the formalism of CTRW or FBM,^36^–^39^ giving interesting theoretical insights into these mechanisms. Nevertheless, these models are most of the time too involved to be applied to experimental data. Thus, in order to relate the simulated and experimental results, we decided to estimate the triplet collision frequency from our simulations and to compare it to the experimental data. Then, using the collision theory, we relate this quantity to the reaction rate constant. Indeed, the collision theory states that the rate constant is proportional to the collision frequency:

6 *k* ∝ *Z*

where *k* is the rate constant and *Z* the collision frequency. Thus, the time dependence of *k* is directly proportional to that of *Z*.

Fig. 3d shows the average number of new molecules that a porphyrin (ground state and triplet) encounters over time, for a full surface coverage (the data were truncated at 100 ns in order to limit finite size effects). The calculation of the new encounter is explained in the “Experimental part” section. Interestingly, the number of new encounter is proportional to *t*^0.87^, which is consistent with the sub-diffusion observed in the simulation and experimental results. The time derivative of the new encounter number is the new encounter frequency and is proportional to *t*^–0.13^. This quantity is the average number of new porphyrins that come in close contact per unit time (here porphyrin means triplet and ground state). The probabilities that the contacting molecule and the considered porphyrins are both triplets is also proportional to the triplet relative concentration. Furthermore, two triplets T* that encounter will react. Thus, if one calls the new encounter frequency *f*, the number of reaction events per unit time, that is the reaction rate, will be:

7 rate = fT^*2^

Identifying this equation with eqn (2) shows that there is a direct equivalence between the rate constant and the new encounter frequency. Thus, as this latter quantity scales proportionally to *t*^–0.13^, the rate coefficient should scale similarly (from eqn (6)). The validity of this approach is confirmed by fitting the full surface coverage decays with the phenomenological reaction rate equation on fractals:^14^,^15^
8
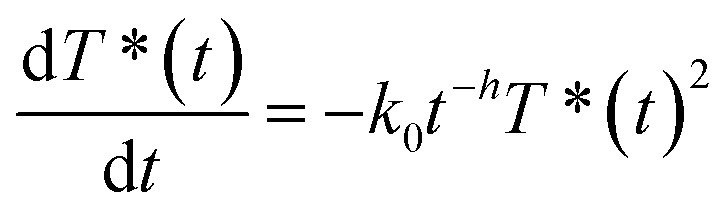



Doing so gives a value of 0.13 ± 0.01 for the *h* parameter, in excellent agreement with the simulated value. Thus, we show that the effects of the complex physics underlying anomalous diffusion can be recovered in a more intuitive expression that provides a phenomenological description of a reaction presenting anomalous kinetics.

## Conclusion

To conclude, we report the first experimental observation of a diffusion-controlled triplet–triplet annihilation reaction in a soft planar environment, and the appearance of an anomalous diffusion regime at high surface converge. This phenomenon is a unique example of a truly diffusion-controlled two-dimensional bimolecular chemical reaction and presents the property of having kinetics that depends on the compactness of the adsorbed molecular layer. At low surface concentrations, the diffusion is Brownian and the reaction can be described with a two-dimensional Smoluchowski model. However, as the concentration increases, the diffusion becomes anomalous and follows a fractional Langevin equation motion. Consequently, the Smoluchowski model fails to adequately account for the experimental results. This behaviour shows similarities with the motion of lipids in biological membranes, and suggests that the anomalous diffusion of these species originates from their high density rather than from a motion in a low-dimensional medium. With the help of molecular mechanics simulations, we have shown that, under simple assumptions, the rate of the TTA could be related to the triplet encounter frequency. This work is an experimental realisation of models usually verified by simulations and opens the way to the exploration of others kinetic schemes, involving for instance macromolecular crowding or obstructed diffusion.

## Experimental part

### Computational details

The molecular mechanics simulations were all carried out using GROMACS 2018.1.^40^–^46^

### Topologies

In order to simplify the topology, the porphyrin that was simulated was the free base TMPyP instead of the metallated ZnTMPyP (the one that is used in the experiments). We believe that this has no influence on the diffusion properties of the molecule.

The charges of the porphyrin core were taken from the coresponding atoms of the OPLS force field, while the charges of the four methylpyridinium ligands were taken from Sambasivarao *et al.*^47^ Doing so, the total charge of the molecule is only 3.32. Thus, the missing charge was evenly dispatched on all the atoms of TMPyP (0.008 charge per atom) in order to reach a total charge of 4. Finally, the polarisation effects at the interface were effectively taken into account by rescaling all the charges of the porphyrin by 0.75 (and counter ions, *i.e.* chloride ion), as suggested by Leontyev & Stuchebrukhov.^48^,^49^ This method provides reliable adsorption behaviour of charged species at liquid–liquid interfaces.^50^ All the bonded parameters were those of the OPLS force field.

The water was simulated using the TIP4P model. The TFT molecules and chloride ions were simulated with the parameters of the OPLS force field.

### Box construction and simulation

The simulated systems consisted in a 15 nm by 15 nm by 7 nm boxes divided in two phases of equal volumes. Periodic boundary conditions were used in all directions. The porphyrins molecules were directly added at the interface. The maximum surface concentration has been measured to be 3.6 × 10^–7^ mol m^–2^ which corresponds to 0.22 molecule per nm^2^. Thus, with the box size of this work, 10% coverage were simulated with 5 TMPyP, 50% with 24 TMPyP and 100% coverage with 48 TMPyP. The total charge of the system was neutralised by addition of four times more chloride atoms than porphyrins molecules. Once constructed, the energy of the boxes was minimised using the steepest descent algorithm until a force threshold of 100 kJ mol^–1^ nm^–1^ was reached. Then, the system was equilibrated for 1 ns, with a time step of 1 fs, in the *NVT* ensemble using the V-rescale thermostat^51^ at 293 K. Finally, the production simulations were carried out in the *NPT* ensemble, using the V-rescale thermostat and the Berendsen barostat^52^ at a temperature of 293 K and pressure of 1 bar, using a time step of 2 fs. During the simulations the LINCS algorithm^53^ was used to constrain all the bonds. Long range dispersion corrections were used for both energy and pressure.

### Adsorption energy

The adsorption energy of the porphyrin was simulated by constructing by umbrella sampling the potential of mean force required to transfer the molecule from the aqueous to the organic phase (see S2†). The starting configurations were generated by placing the molecule under study at the centre of a 4 nm by 4 nm by 4 nm box. The molecule was then solvated in TIP4P water and the box was placed in contact with a 4 nm by 4 nm by 8 nm box, filled with TFT molecules, forming an overall 4 nm by 4 nm by 12 nm box. The energy of the system was then minimized using the steepest descent algorithm until a force threshold of 100 mol^–1^ nm^–1^ was reached. The system was subsequently equilibrated for 1 ns in the *NPT* ensemble. During the equilibration procedure the molecule under study was constrained at its initial position. After the equilibration, the solute was pulled 4.5 nm across the interface, while being constrained in the directions parallel to the interface, still in the *NPT* ensemble. Finally, the potential of mean force was reconstructed using the umbrella sampling technique (g_wham^54^) on several configurations of the solute with respect to the interface. The spacing between two configurations was roughly 0.15 nm along the perpendicular to the interface. However, when necessary, additional configurations were included in order to get a sufficient overlap of the configurations histograms. Each umbrella sampling run was performed for 2.5 ns in the *NPT* ensemble at 293 K and 1 bar using a harmonic biasing force, with a force constant of 2000 mol^–1^ nm^–2^. In the simulation of the transfer the charges of TMPyP were not scaled by 0.75 as in the diffusion simulation, as this is required only to simulate ions at the interface and not in bulk phases.

### Calculation of the new encounter number

The number of new encounter was calculated from the trajectories of the simulations. This quantity represents the total number of porphyrins that came in the neighbourhood of one peculiar porphyrin at a time *t*. However, one molecule cannot be counted two times. The “neighbourhood” being arbitrarily defined by a certain radius. In the present case this radius was set to 1.5 nm, as it is the approximate size of the porphyrin core. The calculation is done as follow. At *t*_0_ and for one porphyrin the number of neighbours is listed. Then, as the simulation proceeds, the neighbours list is updated each time the porphyrin comes closer than 1.5 nm from the center of mass of another porphyrin that is not already in the list. At the end of the simulation the number of elements in the list gives the new encounter number. This number is then averaged over all the porphyrins. Once the new encounter number is known, the reaction rate over the first 100 ns can be estimated by multiplying it by the square of the surface concentration of triplets in the considered time slice. For instance, in the first 100 ns of reaction at full surface coverage, 15% of the molecules are triplets, the new encounter number will thus be multiplied by 0.0225. The obtained value is the number of reaction events on a surface of 225 nm^2^ in 100 ns. It must then be converted to mol m^–2^ by simple arithmetic operations.

## Data availability

The data that support the findings of this study are available from the corresponding author upon reasonable request.

## Author contributions

H. H. G. and G. C. G. conceived the project. G. C. G., A. J. O. and P. F. B. designed the TR-SSHG setup. G. C. G. built up the setup. G. C. G. and M. K. performed the experiments. G. C. G. carried out the simulations and wrote the manuscript. All authors discussed the results and contributed to the manuscript.

## Conflicts of interest

There are no conflicts of interest to declare.

## Supplementary Material

Supplementary information
