# Supplementary material for: Two dimensional diffusion-controlled triplet–triplet annihilation kinetics
Source: Chem Sci. 2019 Jul 4;10(32):7633–40. doi: 10.1039/c9sc00957d (PMC6761882; doi:10.1039/c9sc00957d)
Supplement: Supplementary file 1 [file SC-010-C9SC00957D-s001.pdf]

Electronic Supplementary Material (ESI) for Chemical Science.
This journal is © The Royal Society of Chemistry 2019

Supporting Information: 2D diffusion controlled triplet-triplet annihilation kinetics

Grégoire C. Gschwend,† Morgan Kazmierczak,‡,† Astrid J. Olaya,† Pierre-François Brevet,¶ and Hubert H. Girault*,†

†*Laboratoire d'Électrochimie Physique et Analytique, École Polytechnique Fédérale de Lausanne, Rue de l'Industrie 17, CH-1951 Sion, Switzerland.*

‡*École Normale Supérieure, Département de Chimie, PSL Research University, 75005, Paris, France.*

¶*Institut Lumière Matière, UMR CNRS 5306, Université Claude Bernard Lyon 1, Campus LyonTech La Doua, 10 Rue Ada Byron, 69622 Villeurbanne cedex, France*

E-mail: hubert.girault@epfl.ch

Time-resolved second harmonic generation setup.

The pump and probe beams were generated each by a parametric generator Ekspla PG400 series, pumped by a laser Ekspla PL2230 series. The delay between the pump and probe beams was controlled by a pulse/delay generator BNC model 575. The two beams were collinearly aligned with the help of a non-polarising cubic beam splitter that reflected the probe beam but transmitted the pump. In practice, the pump and the probe beams were not exactly collinear, there was a small angle between them in order to compensate for the dispersion at the glass-liquid interface, ensuring that they were collinear in the cell (see figure S1). Then, they were focused on the interface, in total internal reflection, by a 100 mm lens (spot size ∼100 μm). The second harmonic of the probe beam, the signal, was then collected by a 100 mm lens and sent to a Triax 320 spectrophotometre. A short-pass interference filter cutting the wavelengths longer than 500 nm was placed at the entrance of the spectrophotometre to cut the residuals of the pump beam (565 nm). The signal was then detected by a photomultiplier tube Hamamatsu R928, sent to a boxcar averager and recorded in a computer. The polarisation of the pump and probe pulses were made circular with the help of a quarter-wave plate. Anyway, several combinations of pump and probe light polarisations have been tried and all gave similar lifetimes. The pump pulse energy lied between 0.1 μJ and 2 μJ, the probe energy ranged from 2 μJ to 10 μJ depending on the concentration, both pulses had a duration of ∼30 ps.

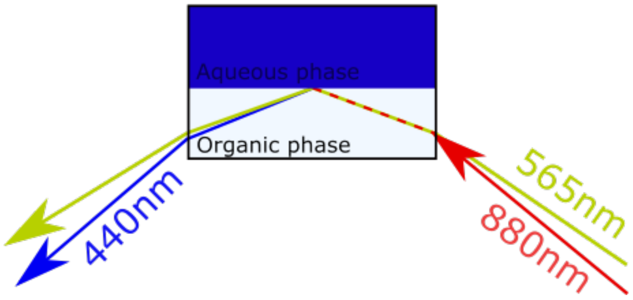

Figure S1: Schematic representation of the pump and probe beams at the interface.

Modus operandi.

Tetrakis(1-methyl-4-pyridinio)porphyrin tetra chloride (ZnTMPyP) was purchased from PorphyChem SAS, α, α, α-trifluorotoluene (TFT) was obtained from Sigma-Aldrich, the water was of Mili-Q quality. All measurements were done in an air-tight optical cell. The cell was prepared in a glove-box under nitrogen atmosphere (concentration of dioxygen 4ppm) and was then placed in an air-tight box equipped with optical windows and kept under a constant nitrogen flux during the measurements. A typical cell was made of 1.5 mL of TFT and 1 mL of aqueous phase containing various concentrations of ZnTMPyP. The cells were prepared at least one hour before the measurements in order to let the interfacial concentration reach its equilibrium value.

The traces were obtained by averaging 250 to 500 measurements. One measurement consists in scanning the pump-probe delay from -0.3 µs to 4 µs by steps of 20 ns (215 steps). Each step being an average over 50 pulses (1 s). Thus, one measurement last 4 minutes, and consequently getting one trace required 15 to 30 hours. As the second-harmonic intensity depends on the square of the surface concentration of adsorbed species, all the traces presented in this work are the square-root of the measured data.

Surface *vs.* bulk concentration.

The surface concentration of ZnTMPyP as a function of the bulk concentration has been measured by surface second harmonic generation as follow . A custom cell of $\sim$ 20 cm^3 was filled with 6.5 cm^3 of TFT and 6.5 cm^3 of water. Then, the concentration of ZnTMPyP was varied by addition of the appropriate volume of a 100 µM solution of ZnTMPyP. For each concentration, the intensity of the second harmonic signal of an incoming 880 nm pulse, resonant with the Soret band of ZnTMPyP, was measured during 20 minutes (average over

$6{\cdot}10^4$ pulses) after $\sim$ 1 h of equilibration, increasing the concentration from 500 nM to 50 µM. This procedure was repeated five times. The data have been fitted assuming a Langmuir isotherm (figure S2a):

$$\frac{\Gamma(c)}{\Gamma_{max}} = \frac{c\beta}{1 + c\beta} \tag{1}$$

where $\Gamma(c)$ is the surface concentration of ZnTMPyP, Γ_{max} the maximum surface concentration, c the bulk concentration and β the Langmuir parameter. A Frumkin isotherm did not approprietly fit the experimental data, which implies that the interaction between the adsorbed molecules is weak. The value found for the adsorption Gibbs free energy is $-31.0{\pm}0.3$ kJ·mol^{-1}, which indicates a strong affinity of the porphyrin for the interface. This value is in concordance with that obtained by molecular dynamics, $-33{\pm}1$ kJ·mol^{-1}, as well as with previously published values.[S1] Using the experimental results, the surface coverage of ZnTMPyP at bulk concentrations used in this work, 500 nM, 5 µM and 50 µM, is estimated to be 12±1%, 58±1% and 93±1%.

Maximum surface concentration.

The maximum surface concentration has been obtained by measuring the surface tension of the water-TFT interface as a function of the bulk concentration of ZnTMPyP by the pendant drop method. A drop of TFT was formed at the tip of a glass pipette in a quartz cuvette filled with Mili-Q water saturated with TFT. The surface tension was obtained by fitting of the curvature of the solvent drop. The curvature measurement and fitting were done on a Kruss DSA-30 drop shape analyser. As in the case of the isotherms, the concentration of ZnTMPyP was varied by addition of the appropriate volume of a 100 µM solution of ZnTMPyP. The values were reported after $\sim$ 1 h of equilibration. The data have been fitted using the Gibbs adsorption equation, assuming again a Langmuir isotherm (figure S2b). Thus, the maximum surface coverage was found to be $3.6{\cdot}10^{-7}$ mol·m^{-2}, which implies a molecular surface of $\sim$4.6 nm^2, in good concordance with the approximate size of ZnTMPyP, *i.e.* a square of 2 nm

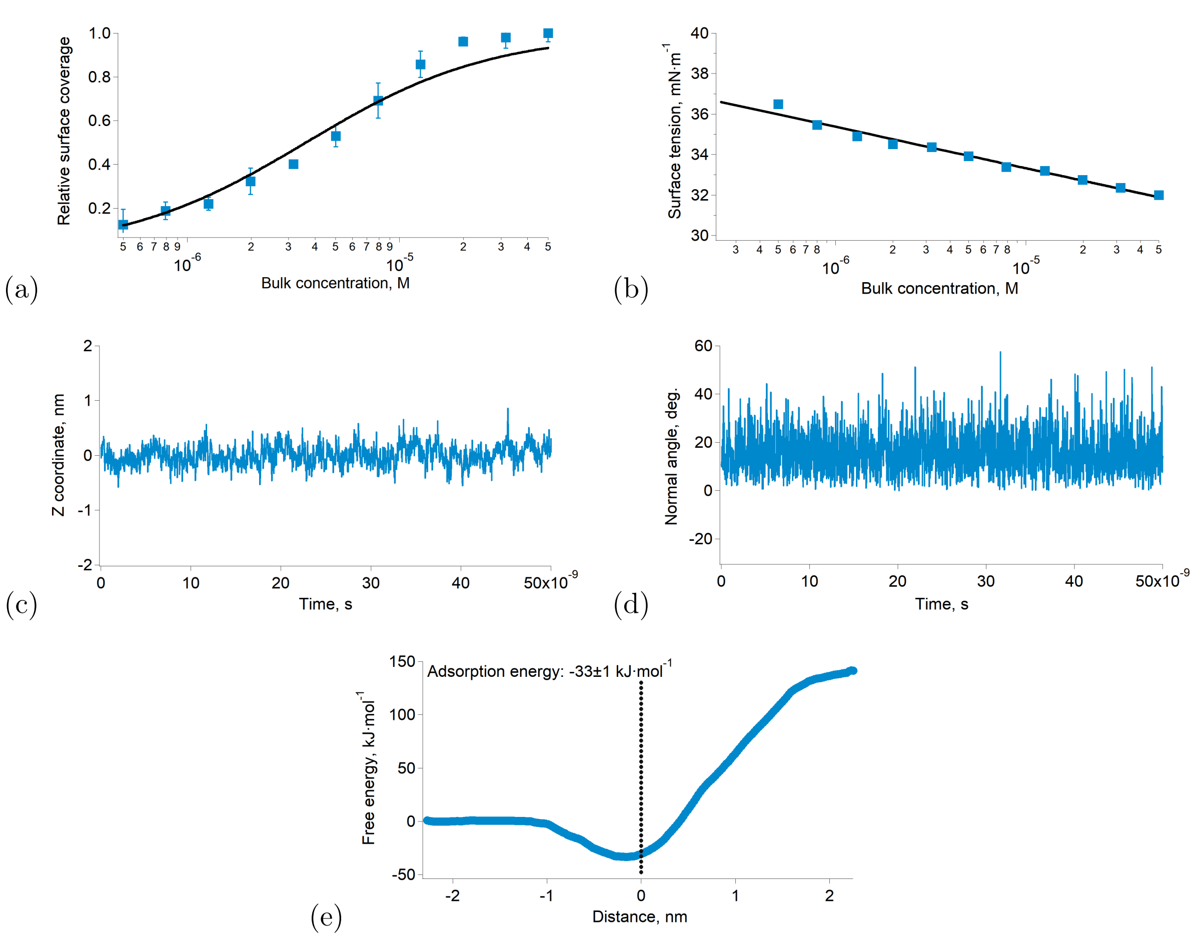

Figure S2: **Adsorption properties of the porphyrin at the water-TFT interface. a)** Adsorption isotherm of ZnTMPyP at the water TFT interface (mean values and standard deviations). The concentration of adsorbed molecule was measured by SHG. The data were fitted assuming a Langmuir isotherm $\Delta G_{ads} = -33.0 \pm 1$ kJ·mol^{-1}. **b)** Surface tension of the water-TFT interface as a function of the bulk concentration of ZnTMPyP measured by the pendant drop method (mean values, standard deviations smaller than the symbols). The data have been fitted using the Gibbs adsorption equation and a Langmuir isotherm $\Gamma_{\mathrm{max}} =$ 3.6±0.2·10^{-7} mol·m^{-2}. **c)** z coorpodinate (perpendicular to the interface) over the course of the simulation of the center of mass of one TMPyP moiety simulated by molecular mechanics. **d)** Normal angle over the course of the simulation of the center of mass of one TMPyP moiety simulated by molecular mechanics. **e)** Free energy profile of TMPyP across the water-TFT interface simulated by molecular mechanics. The dashed line shows the interface position.

sides. Consequently, at full surface coverage, the adsorbed molecules form a closely packed film.

Porphyrin orientation at the interface.

The coordinate perpendicular to the interface of the centres of mass of the pophyrins was found to be stable over time, with a standard deviation of 180 pm (figure S2c), meaning that the reaction is effectively taking place in a two dimensional environment, altough the interfacial capillary waves tends to roughen the surface on a larger scale.[S2] The simulations also show that the molecules are slightly tilted on the interface with an average normal angle of $16° \pm 8°$ (S2d), in agreement with the previously reported value.[S1] Assuming an extreme value of 24° and a lenght of 1.5 nm for the porphyrin, the z-coordinates of the highest and lowest atoms is 0.6 nm. Thus, adding the fluctuations of the centre of mass and that due to the tilting, the total variation of the z-coordinates is of the order of 0.8 nm.

Triplet spectrum and aggregation

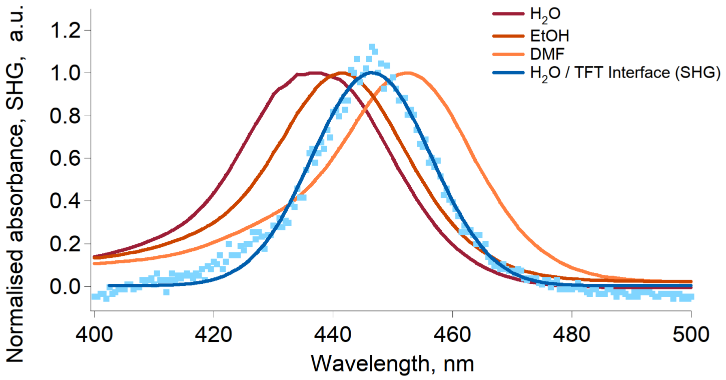

Figure S3: **ZnTMPyP spectrum at the liquid-liquid interface.** Normalised UV-vis and SHG spectra of ZnTMPyP in various solvents. The concentrations were 10 μM for the UV-vis spectra and 100 μM for the SHG spectrum. It can be seen that there is a red shift of the spectra with decreasing polarity of the solvents ($E_T{}^N = 1$, 0.654, 0.386, 0.241 for H_2O, EtOH, DMF and TFT.[S3] Interface $E_T{}^N$: ~ 0.620[S4–S6]). We explain the shift at the interface by solvatochromism rather than by aggregation.

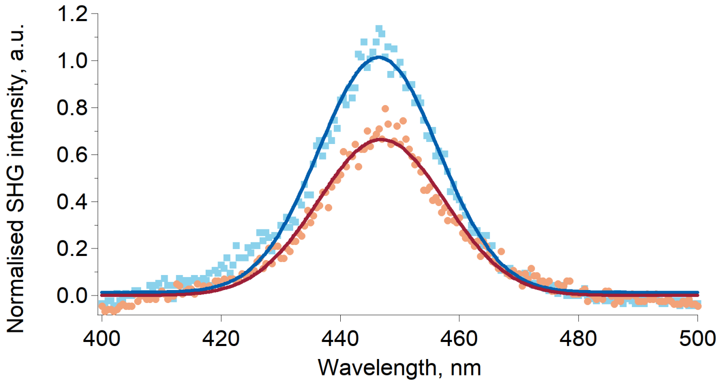

Figure S4: **ZnTMPyP spectrum under excitation.** ZnTMPyP SHG spectrum without (blue squares) and with (brown circles) excitation at 565 nm (energy per pulse 1 μJ). The excited spectrum was recorded 100 ps after the excitation.

Data fitting with the two-dimensional diffusion-controlled kinetic model

Theory

The second-harmonic generation data have been fitted with the two-dimensional diffusion-controlled kinetic model derived by Razi-Naqvi[S7] and by Owen.[S8] This model relies in the resolution of the Fick's second law of diffusion in cylindrical coordinates proposed by Carslaw and Jaeger.[S9] The model is that of an infinite plane with a circular hole of radius ρ centred at the origin. The initial concentration if c_0 everywhere in the plane at negative times (except in the hole). At $t \geq 0$ the concentration on the circumference of the hole is 0. Thus, the equation to solve is:

$$\frac{\partial c}{\partial t} = D\left(\frac{\partial^2 c}{\partial r^2} + \frac{1}{r}\frac{\partial c}{\partial r}\right) \tag{2}$$

with the boundary and initial conditions:

$$c(r = \rho, t > 0) = 0 \tag{3}$$

$$c(r > \rho, t = 0) = c_0 \tag{4}$$

The solution of this equation is:

$$c(r,t) = \frac{2c_0}{\pi}\int_0^{\infty} \exp\left(-Du^2 t\right)\frac{J_0\left(u\rho\right)Y_0\left(ur\right) - J_0\left(ur\right)Y_0\left(u\rho\right)}{u\left(J_0^2\left(u\rho\right) + Y_0^2\left(u\rho\right)\right)}du \tag{5}$$

Equation 5 gives the concentration of an infinite system of initial concentration c_0 consisting of a sink of radius ρ "opened" at $t = 0$. One of the properties of Equation 5 is that $\lim_{r\to\infty} c(r,t) = c_0$. This is a consequence of the initial condition 4.

Equation 5 can be used to obtain the rate of a two-dimensional fluorescence quenching experiment. Indeed, the reaction rate is given by the flux of quencher (of initial concentration Q_0) towards a fluorophore of radius ρ times the fluorophore concentration $F(t)$. The quencher

flux is thus:

$$\begin{aligned}\phi(r,t)_{r=\rho} &= 4\pi D N_A \left(r\frac{\partial Q}{\partial r}\right)_{r=\rho}\\ &= \frac{16DQ_0N_A}{\pi}\int_0^\infty \frac{\exp\left(-Du^2t\right)}{u\left(J_0^2\left(u\rho\right)+Y_0^2\left(u\rho\right)\right)}du\end{aligned} \tag{6}$$

and for a fluorophore having a long-lived excited state (*i.e.* the first-order decay can be neglected), the kinetic of the reaction is:

$$\frac{dF(t)}{dt} = Q_0F(t)\frac{16DN_A}{\pi}\int_0^\infty \frac{\exp\left(-Du^2t\right)}{u\left(J_0^2\left(u\rho\right)+Y_0^2\left(u\rho\right)\right)}du \tag{7}$$

In this case, it is assumed that the quencher concentration is much larger than that of the fluorophore and that the quenching is only limited by the diffusion (*i.e.* that the reaction takes place as soon as the reagents are close to each others). Therefore, the property that $\lim_{r\to\infty} Q(r,t) = Q_0$ can apply. This situation is schematically represented in Figure S5.

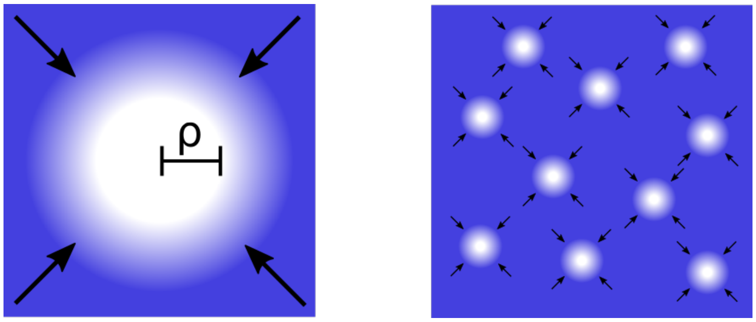

Figure S5: **Scheme of a two-dimensional diffusion controlled fluorescence quenching.**The quencher concentration is represented in blue, the arrows show the direction of the flux. The situation on the right show the case where many fluorophores are in the reaction medium. It is assumed that the concentration between the fluorophores is Q_0.

In the present article, we adapted the same method to the triplet-triplet annihilation kinetics. However, in this case the quencher concentration no longer tends to a constant value far from the fluorophore because the fluorophore and quencher are the same molecules. Nevertheless, since in Equation 6 the quencher concentration at infinite distance appears as a factor in front of the integral, we replace it by $q(t)$ in the case of TTA. Thus we do the

following substitutions,

$$f(t) \to T(t)$$

$$Q_0 \to T(t)$$

Consequently, Equation 7 becomes:

$$\frac{dT(t)}{dt} = T(t)^2 \frac{16DN_A}{\pi} \int_0^\infty \frac{\exp\left(-Du^2t\right)}{u\left(J_0^2\left(u\rho\right) + Y_0^2\left(u\rho\right)\right)} du \tag{8}$$

Fitting code

The diffusion coefficient of the triplet was obtained from the normalised triplet concentrations decay traces. The normalisation implies that:

$$T_n(t) = \frac{T(t)}{T_0} \tag{9}$$

$$\frac{dT_n}{dt} = \frac{1}{T_0}\frac{dT(t)}{dt} \tag{10}$$

Thus, Equation 8 becomes:

$$\frac{dT_n(t)}{dt} = T_0 T_n(t)^2 \frac{16DN_A}{\pi} \int_0^\infty \frac{\exp\left(-Du^2t\right)}{u\left(J_0^2\left(u\rho\right) + Y_0^2\left(u\rho\right)\right)} du \tag{11}$$

The core of the MATLAB fitting code is then:

%Options of the non-linear fitting and differential equation solver.

options = optimoptions(@lsqcurvefit, 'UseParallel', true, 'OptimalityTolerance', 1e-10, 'FunctionTolerance', 1e-10, 'StepTolerance', 1e-10);

optionsODE = odeset('Reltol', 1e-13, 'AbsTol', 1e-14);

%Starting value, lower bound and upper bound of diffusion coefficient.

startingVals = 1e-12;

lb = 1e-14;

ub = 1e-6;

%Deffinition of the fitting model. T is the normalised concentration, p the diffusion coefficient, tspan the time and G_triplet the normalisation constant.

```
function T = model(p,tspan)
function dTdt = ode(t,T)
dTdt = (-16 * 6.022e23 * G_triplet * p(1)/pi) * integral ( @(u) exp(-p(1) * u.^ 2 * t/ (1.5e-9)
^2) / (u.*(besselj(0,u).^2 + bessely(0,u).^2)), 1e-10, 10000, 'ArrayValued', true) * T^2;
end
[t,T] = ode113(@ode, tspan, p_TTA(1,1), optionsODE);
end
[p, resnormp, residp, exitflagp, outputp, lambdap, Jp] = lsqcurvefit(@ model, startingVals,
tspan, Data, lb, ub, options);
```

%Calculation of the confidence interval:

```
cip1p=nlparci(p, residp, 'jacobian', Jp)
```

%Plot of the fitting

```
fit_2(:,1)=-model(p,tspan);
plot(tspan, fit_2(:,1));
```

References

(S1) Nagatani, H.; Piron, A.; Brevet, P.-F.; Fermin, D. J.; Girault, H. H. Surface Second Harmonic Generation of Cationic Water-Soluble Porphyrins at the Polarized Water|1,2-

Dichloroethane Interface. *Langmuir* **2002**, *18*, 6647–6652.

(S2) Luo, G.; Malkova, S.; Yoon, J.; Schultz, D. G.; Lin, B.; Meron, M.; Benjamin, I.; Vanýsek, P.; Schlossman, M. L. Ion Distributions near a Liquid-Liquid Interface. *Science* **2006**, *311*, 216–218.

(S3) Reichardt, C. Solvatochromic Dyes as Solvent Polarity Indicators. *Chemical Reviews* **1994**, *94*, 2319–2358.

(S4) Wang, H.; Borguet, E.; Eisenthal, K. B. Polarity of Liquid Interfaces by Second Harmonic Generation Spectroscopy. *The Journal of Physical Chemistry A* **1997**, *101*, 713–718.

(S5) Wang, H.; Borguet, E.; Eisenthal, K. B. Generalized Interface Polarity Scale Based on Second Harmonic Spectroscopy. *The Journal of Physical Chemistry B* **1998**, *102*, 4927–4932.

(S6) Ishizaka, S.; Kim, H.-B.; Kitamura, N. Time-Resolved Total Internal Reflection Fluorometry Study on Polarity at a Liquid/Liquid Interface. *Analytical Chemistry* **2001**, *73*, 2421–2428.

(S7) Naqvi, K. R. Diffusion-controlled reactions in two-dimensional fluids: discussion of measurements of lateral diffusion of lipids in biological membranes. *Chemical Physics Letters* **1974**, *28*, 280 – 284.

(S8) Owen, C. S. Two dimensional diffusion theory: Cylindrical diffusion model applied to fluorescence quenching. *The Journal of Chemical Physics* **1975**, *62*, 3204–3207.

(S9) Carslaw, H. S.; Jaeger, J. C. Some Two-Dimensional Problems in Conduction of Heat with Circular Symmetry. *Proceedings of the London Mathematical Society* **1940**, *s2-46*, 361–388.
